# Integrating Public Health and Health Promotion Practice in the Medical Curriculum: A Self-Directed Team-Based Project Approach

**DOI:** 10.3389/fpubh.2017.00193

**Published:** 2017-08-21

**Authors:** Geraldine Kershaw, Michal Grivna, Iffat Elbarazi, Souheila AliHassan, Faisal Aziz, Aysha Ibrahim Al Dhaheri

**Affiliations:** ^1^Medical Education Department, College of Medicine and Health Sciences, United Arab Emirates University, Al Ain, United Arab Emirates; ^2^Institute of Public Health, College of Medicine and Health Sciences, United Arab Emirates University, Al Ain, United Arab Emirates

**Keywords:** health promotion, health education, undergraduate public health education, self-directed learning, student projects, health professions education research, project-based learning

## Abstract

Preparing health professionals in health promotion (HP) and disease prevention is essential for improvement of population health, community HP, and better health care for individuals. The aim of this article is to describe an HP project in the form of a major self-directed project-based learning task integrated within the curriculum in the second year of the medical degree program at United Arab Emirates University. The project introduces students to public health and HP practice and develops students’ literature searching, writing, presentation skills, and team work. Students learn the principles underlying behavioral change, and the design of HP programs and materials, through a lecture format. Small groups of students each choose a specific health topic for their project. Over 11 weeks, students obtain information about their topic from appropriate sources (library, PubMed, Google Scholar, credible health sources such as World Health Organization). Using the principles learned in the lectures, they develop appropriate materials for their target audience: for example, posters, a pamphlet, social media content, or a video or radio message. Students seek advice from specialist faculty as needed. In week 12, each team presents their project background, rationale, and materials to their colleagues in a seminar format open to all faculty. They then submit the materials they developed for assessment. Group marks are assigned for presentations and materials. Key concepts are assessed by multiple choice questions in comprehensive course examinations. By participation in the HP project, many students develop a solid background in prevention. The information retrieval, writing, and presentation skills, as well as experience of team work, are valuable both for the remaining years of their training and their future careers.

## Background and Rationale for the Educational Activity

Preparing medical professionals in health promotion (HP) and disease prevention is considered an essential step for community HP, better health care for individuals, and development of population health strategies (1–5). The World Federation for Medical Education identified HP as one of its priorities in the Edinburgh Declaration (6). The World Health Organization and others identified the training of health-care providers in public health and community medicine as a key measure for better health care and for improving population health (4, 7–9), yet in the last century, courses in HP and prevention in the medical curriculum were patchy and unsystematic (3); they gained more attention only recently (10–14).

Garr et al. suggest that prevention teaching should be a priority and should be incorporated throughout the medical curriculum under the supervision of specialized faculty responsible for monitoring its content and quality (1). Studies have reported that not only do medical students begin their program with poor knowledge about healthy lifestyle and prevention, but also that their health, their personal lifestyle, and their perceptions about the importance of preventive approaches all deteriorate during their studies (11, 12, 15, 16). Such reports have raised fears that future health-care providers may contribute to worsening HP and public health practice, both by their lack of knowledge and skills in health education and promotion practice, and by being negative role models (16, 17). In the United Arab Emirates (UAE), a fast developing country in the Middle East, chronic diseases and lifestyle-related health problems are increasingly placing a burden on the health-care system (18–24), hence it is particularly important to address these issues in the medical curriculum.

The United Arab Emirates University (UAEU) offers an HP course component in the second year of the medical degree. This builds on content and skills developed through a self-directed lifestyle project earlier in the program. Lifestyle related elements were introduced to the medical curriculum for first year medical students at UAEU in 2001, reported previously by Barss et al. (16). The authors recommended that more training and education related to public health medicine should be part of the medical curriculum to ensure graduates are well prepared to meet national health priorities, in addition to improving students’ own health and practice regarding lifestyle and disease prevention. A self-directed HP project was also included in the curriculum for first year students in 2001. Fundamental changes to the curriculum from 2011 took the HP project to its current format in the second year of the curriculum. To the best of our knowledge, no other universities in the UAE or the region offer a self-directed HP project in the medical curriculum that aims to educate students on both knowledge and skills relevant to this topic.

The aim of this article is to describe this HP project, a major self-directed learning task integrated within the curriculum in the second year of the medical program in UAEU. The project introduces students to public health and HP practice and requires application of literature searching, writing, presentation skills, and team work.

## The Pedagogical Framework and Pedagogical Principles Underlying the Educational Activity

The course element that is described here, the HP project, is in the form of a project component in a one semester course entitled Professional Practice and Communication 3 (PP&C3), one of four consecutive one semester PP&C courses in the first 2 years (PreMed) of the degree program. These four courses introduce concepts in medical practice, while at the same time equipping students with problem-solving and analytical tools as well as communication skills for research and professional practice. In PP&C3, content for medical practice includes theoretical underpinnings of HP, health education, and interventions focused on population health, as well as topics in ethics and professionalism.

Project-based learning falls in the broad field of active learning. The benefits of project-based learning are explained by social cognitive and constructivist learning theories (25). Project-based learning is similar to problem-based learning in that learning is driven by problem-solving, but the production of an artifact by a collaborative student team links it firmly to activity theory (26). Helle et al. posit six key features of project-based learning in higher education relevant to cognitive psychology and the promotion of effective learning (25). The project described attempts to comply with all of these.

The first key feature is problem orientation: a specific question encourages a search for knowledge and the development of expertise. Our students must develop their understanding of theory and practice of HP to develop their project. Second, project-based learning requires students to create a concrete artifact, but to do this they must not only learn subject matter: they must also apply generic skills such as time management and team work. Production of HP materials while working in independent teams clearly fulfils this criterion. Third, students are in control of their own learning, deciding for themselves how to solve project-related problems. Specialist faculty are available to support our student teams but do not intervene unless essential. Fourth, projects are commonly situated in an authentic context. This is the most problematic area for our HP project, because although all materials must be designed for the local context there are limited opportunities to test them. Fifth, projects may require several formats for communication of products, and this is built in to our project, students being required to produce not only the artifact in the form of HP materials but also a progress report and a group oral presentation. Finally, projects may stimulate students to take responsibility for their own learning and improve positive motivation. The analysis of our student questionnaire about the project attempts to establish the extent to which this was achieved.

## Competencies Underlying the Project as Related to Learning Objectives

The MD program at CMHS has six program learning outcomes that students must achieve, related broadly to knowledge and its application to the patient; communication; patient care; lifelong learning; professionalism; and the health-care system. HP project is particularly relevant to PLO 2, “Demonstrate communication skills that are effective in the exchange of information and collaboration with patients, their families, and health professionals,” but it also supports development of competency in skills for lifelong learning, knowledge development, and professionalism. Course learning objectives (CLOs) for the PP&C3 course as set out in the course description include that students should be able to: (1) critically evaluate published research; (2) describe the ethical implications of conducting research involving animals and humans; (3) apply models of behavioral change and principles of HP and education to design a project for HP; (4) work effectively in teams to produce an effective HP project for a target group; (5) identify types of argument in formal oral contexts (scientific presentations and debates); (6) explain professional, ethical, and moral issues related to best medical practice and scientific endeavor; (7) demonstrate in writing the critical reflective skills essential to being a reflective practitioner; and (8) apply appropriate communication skills for a range of audiences and communicative needs.

The HP project aims specifically to promote achievement of objectives 3, 4, and 8 through a productive team project, which requires students to use appropriate communication skills to demonstrate understanding of how HP theories work in practice. Students also need to show they are developing the professional attributes to work in a team and demonstrate communication skills, both written and oral. The design of the HP project integrates the teaching and assessment of these concepts; in addition, it requires students to apply research skills and critically appraise articles (CLO 1) to research the background and epidemiology of their chosen HP topic.

## Learning Environment (Setting, Students, and Faculty)

The student population consists of second year students in a 6-year undergraduate program at the College of Medicine & Health Sciences, UAEU, in Al Ain, UAE. All students are UAE nationals, first language Arabic; the language of instruction is English. Some students read slowly and have poor research skills, hence benefit from learning tasks which explicitly require them to read. Male and female students are taught separately, and outside the educational context their lives are quite different: the males are free to leave the hostel when they wish and can easily meet outside class, whether for study or social reasons, but some females have less social freedom than others and cannot leave the hostel or meet colleagues who live elsewhere outside of class hours. Those faculty members involved in the teaching for this project are specialists in related topics such as HP, public health and education, and communication skills. Visiting faculty include specialists from the local Ministry of Health (Health Authority Abu Dhabi) and a health education professional from a nearby hospital.

## Pedagogical Format

The HP project is a group task completed over the first 10 teaching weeks of a one semester course. The Course Coordinator assigns students to teams of five to seven people (depending on cohort or class size). Students are ranked according to their scores in research essay writing and presentation skills in the first year of the program, as these scores are predictors of ability in speaking, reading, and writing, all essential for the project. Then, the top students will all go to different teams and so on to ensure that all teams have a mix of abilities. They participate in a team building exercise (entitled Lost at sea) during the first week of classes (27), and then for the whole semester are required to sit with their teams for class activities, both those directly related to the project and other learning tasks, to encourage a team ethos. Some students complain about being in assigned groups rather than friendship groups, so a brief discussion of the importance of team work in the medical context is included.

We then explain the project to the students. We first clarify the rationale behind teaching HP content and explain they will develop team work skills as well as research and communication skills through application of HP theory to a topic of their own choice. Discussion of key public health issues at this stage heightens student awareness of possible topics.

For the project, students must identify a target health behavior and explain why it is a health problem; identify a target group for intervention; identify the behavior change goal; develop an action plan and tools (e.g., pamphlets, posters, videos, and audio files) for their target audience; and finally submit HP material for evaluation by faculty from the Institute of Public Health (IPH) and present their material to colleagues and faculty orally. All CMHS faculty and students are invited to attend the oral presentations.

Key concepts in HP are taught in lecture format by IPH Faculty and specialist staff from the Ministry of Health and a hospital health education department. Topics include theory of HP and education; models of behavioral change; HP planning; HP needs; HP tools development; focus groups in research and health education; and HP evaluation. Critical appraisal and analysis of journal articles, medical ethics, and research ethics are taught in lecture, group work and discussion format by faculty from the Departments of Medical Education and Family Medicine, using a range of materials as appropriate. Development of problem-solving and communication skills is tackled through the design of the HP project: to encourage application of problem-solving and analytical skills students are required to put HP theory into practice while working in their teams to develop locally relevant HP materials.

Table 1 shows the basic schedule of activities, both in and out of timetabled class time, and deadlines.

**Table 1:** Project elements: timing and team work.

Week	Lectures	Students self-directed work	Skills development	Assessment/comments
Week 1	Introduction to health promotion (HP)	Teams assigned by faculty	Team formation and team work	Teams of 5–8 students

Weeks 2–4	Theory and models in HP HP needs assessment	Team building Literature search for health priority	Team work Literature search and journal reading	Specialist faculty available for advice

Week 5	HP planning Focus groups—theory and practice	Selection of HP topic and target population	Clarifying HP messages	Topic submission, approval by faculty to match national public health priorities

Weeks 6–9	Models of behavioral change HP tools development	Further background reading	Team work Tools development	

Week 7	Focus groups—theory and practice	Further background reading Team work on tools and materials	Team work Tools development	Formative feedback on simulated task

Week 8	Communicating health information HP evaluation	Team work on tools and materials	Use of social media Testing materials	

Week 9	Clarifying HP messages	Progress report	Progress report writing and submission	Assessment of progress report

Weeks 10–11		Editing materials	Group oral presentation skills Editing and review	Week 10: midterm examinations (Individual Marks)

Week 12		HP oral presentation Submission of all tools/materials		Presentations are open to all faculty and assessed for both content and presentation skills (group marks) public health and HP specialists assess the tools (group marks)

Week 13		Reflective writing	Reflection and critical appraisal	Reflection on teamwork confirms development of professionalism, clarifies areas for improvement

Students are expected to complete the bulk of project-related work out of class, but some scheduled class time is also devoted to project work, facilitated by Medical Education staff who ask and answer questions. This is particularly necessary for female students for the reasons described earlier. Students are regularly reminded that sufficient reading of appropriate sources must underpin choice of topic and target audience, and there are class sessions devoted to developing skills for selecting and reading research articles. These skills are introduced in the first semester of the PreMed program but developing proficiency takes time. Teams are free to choose their own topics, but oral discussion of topics with team members before approval by the Course Coordinator in week 5 ensures that students have done sufficient background research, and also that all team members are in agreement. Once a broad topic has been approved, teams do further reading and are also encouraged to discuss their topic with appropriate clinical faculty.

*Focus group practice* is a simulation led by an HP professional from the neighboring hospital: project teams play the role of focus group members, evaluating extant locally produced HP materials. *Progress reports* follow a standardized format, requiring teams to answer specific questions about their project objectives and design, and are completed in class on paper to encourage full team participation in the drafting of responses. This task is particularly helpful for teams who may have been slow to get started on developing materials: some of our undergraduates struggle with time management.

*The oral presentation* (week 12) must include discussion of the importance of the health topic addressed; details of the target group addressed in their health message and why they were chosen; explanation of the model and theory of behavioral change selected; description and demonstration of the type of materials developed, including rationale, goal, and specific messages in the materials; strengths and weaknesses of the materials; and recommended modalities for evaluation of the impact of the project on knowledge, attitudes, and practices of the target group. All members of the team must speak, and all team members get the same mark, so students are encouraged to practice together and to coach those students with weaker oral skills. 20 min is allocated for each team, including time for questions. Medical Education faculty assess each team on communication skills and team work. IPH faculty assess content of presentations as well as quality of materials. The HP project forms a key component of assessment for the course (20%). Key HP concepts are also assessed by MCQs in course examinations.

## Results to Date/Assessment (Processes and Tools; Data Planned or Already Gathered)

Impact of the project on students’ understanding of HP, health education, and lifestyle and health was assessed after completion of the projects by a self-administered hard copy questionnaire for each cohort 2013–2015 (*n* = 206). The questionnaire focused primarily on students’ perception of impact of the HP project on their knowledge and skills development in research skills, understanding and application of HP, team work, and communication skills. There were 37 statements which students assessed using a 4-point Likert scale, where 1 = strongly agree; 2 = agree; 3 = disagree; and 4 = strongly disagree. The questionnaire also included 14 yes/no questions about any changes students had made in their personal lifestyle as a result of the HP project. The data on those questions are not analyzed herein.

**Figure 1:**
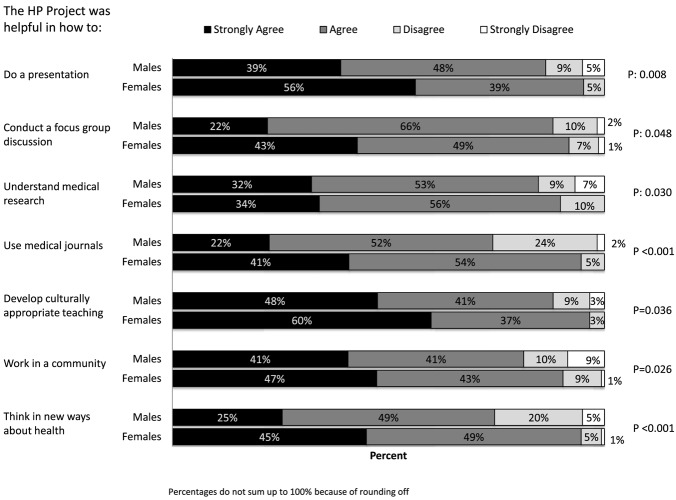
Students’ assessment of the impact of health promotion project, by gender (*n* = 206).

The data concerning HP project impact were entered into Statistical Package for Social Sciences version 21 and then transferred to Stata version 14 for statistical analysis (28, 29). Overall student evaluations of the statements were tabulated as frequencies and percentages under four themes: communication skills; team work; understanding and application of HP concepts; and research process and academic integrity (Table 2). Chi-square tests and Fischer Exact tests were applied to compare responses of male and female students, and those data are presented as a clustered bar chart (Figure 1).

**Table 2:** Students’ evaluation of health promotion (HP) project (*N* = 206).

Knowledge/skills	Strongly agree	Agree	Disagree	Strongly disagree
	*n* (%)	*n* (%)	*n* (%)	*n* (%)
**The HP project**				
**1. Research process and academic integrity**				
Helped in learning how to search medical/scientific literature	89 (43.4)	105 (51.2)	11 (5.4)	0 (0.0)
Helped in learning how to use research journals for future medical practice	73 (35.6)	109 (53.2)	22 (10.7)	1 (0.5)
Helped me to understand research in medical journals	69 (33.5)	113 (54.9)	20 (9.7)	4 (1.9)
Encouraged me to conduct future research in public health	43 (21.0)	99 (48.3)	50 (24.4)	13 (6.3)
Helped in learning how to prepare for future evidence-based practice	74 (36.1)	107 (52.2)	21 (10.2)	3 (1.5)
Helped in learning how to protect my future patients and populations	62 (30.1)	116 (56.3)	22 (10.7)	6 (2.9)
**2. Understanding and application of HP concepts**				
Helped in learning how to use different models of HP	94 (45.6)	95 (46.1)	16 (7.8)	1 (0.5)
Stimulated interest in public health and community medicine	60 (29.3)	101 (49.3)	36 (17.5)	8 (3.9)
Motivated me to have more teaching in public health	48 (23.3)	86 (41.8)	60 (29.1)	12 (5.8)
Improved my perceptions about public health	63 (30.7)	114 (55.6)	24 (11.7)	4 (2.0)
Stimulated me to think in new ways about health	68 (33.3)	100 (49.0)	32 (15.7)	4 (2.0)
Helped me to understand the importance of prevention	87 (42.4)	98 (47.8)	17 (8.3)	3 (1.5)
Helped in learning how to promote healthy lifestyles in my community	94 (45.6)	94 (45.6)	16 (7.8)	2 (1.0)
Helped in learning how to work with patient education	65 (31.5)	113 (54.9)	23 (11.2)	5 (2.4)
Helped in learning how to work in a community	93 (45.4)	87 (42.4)	19 (9.3)	6 (2.9)
Helped in learning how to incorporate insights from a focus group into HP materials	51 (25.0)	122 (59.8)	31 (15.2)	0 (0.0)
Helped in learning how to develop culturally appropriate materials for a United Arab Emirates target group	116 (56.3)	78 (37.9)	10 (4.8)	2 (1.0)
Helped in learning how to develop attractive and appealing designs for a target audience	101 (49.0)	92 (44.7)	10 (4.8)	3 (1.5)
Helped in learning how to develop clear and simple HP messages for laypersons	102 (49.8)	97 (47.3)	6 (2.9)	0 (0.0)
Helped in learning how to choose appropriate media vehicle(s) for different HP purposes	116 (56.8)	85 (41.7)	3 (1.5)	0 (0.0)
Helped in learning how to select a target population for a health issue	116 (56.3)	87 (42.2)	3 (1.5)	0 (0.0)
Helped me to understand how to conduct a focus group interview	76 (36.9)	111 (53.9)	16 (7.8)	3 (1.5)
Helped in learning how to use a focus group to evaluate materials	78 (37.9)	110 (53.4)	18 (8.7)	0 (0.0)
Helped in learning how to evaluate HP interventions using pre- and posttesting on KAP	74 (35.9)	110 (53.4)	21 (10.2)	1 (0.5)
**3. Working in teams**				
Helped in learning how to work in a team	108 (52.7)	72 (35.1)	16 (7.8)	9 (4.4)
Helped in learning how to collaborate with other organizations	39 (18.9)	101 (49.0)	50 (24.3)	16 (7.8)
**4. Written and oral communication skills**				
Helped in learning how to develop my communication skills	60 (29.1)	123 (59.7)	19 (9.2)	4 (1.9)
Helped in learning how to do a good presentation	106 (51.4)	85 (41.3)	12 (5.8)	3 (1.5)
Helped me to understand the importance of communication with patients/community	80 (38.8)	106 (51.5)	18 (8.7)	2 (1.0)

*n: frequency*.

*Frequencies may not always add up to total sample size due to missing values. Percentages do not sum up to 100% because of rounding off*.

**Figure 2:**
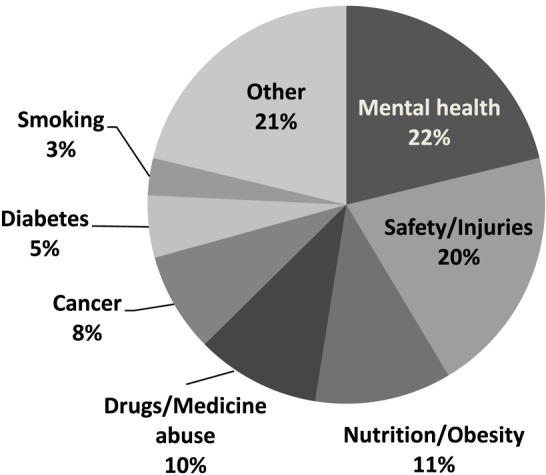
Topics of health promotion projects 2013–2015 (*n* = 61).

**Figure 3:**
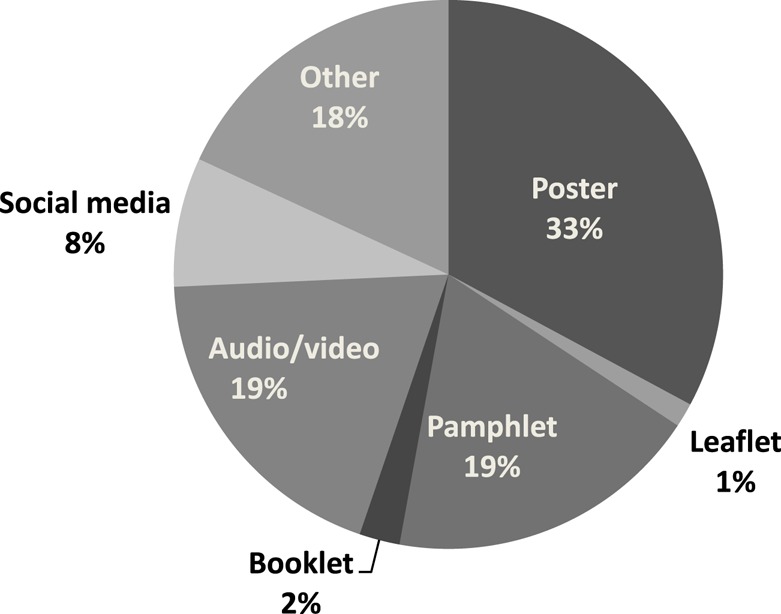
Health promotion tools developed by type 2013–2015 (*n* = 210).

Overall response rate for the questionnaire was 57.1% (206/361). A higher proportion of males [75.6% (59/78)] responded than females [51.9% (147/283)]. The response rates in 2013, 2014, and 2015 were 45.1% (60/133), 72.1% (93/129), and 53.5% (53/99) respectively. Lower response rates may be related to timing of questionnaire distribution. Topics of student projects for 3 cohorts of students (61 projects) were categorized to identify themes (Figure 2). Types of HP materials (posters, leaflets, etc.) developed by students were categorized to identify which were most frequently produced (Figure 3).

Overall, the students perceived that HP project had a positive impact on the development of their knowledge and skills. The majority of students strongly agreed or agreed that the HP project helped in developing their skills in communication, team work, and research, and their understanding of and interest in HP concepts (Table 2). A higher proportion of females than males strongly agreed that HP project developed their knowledge and skills. The clustered bar chart shows those areas where there were significant differences between female and male students’ perception of the impact of HP project on their knowledge and skills (Figure 1). Such differences in their strong agreement with the statements were noted in the following areas: how to do a presentation (56% females vs 39% males, *p*: 0.008); how to conduct a focus group discussion (43% females vs 22% males, *p*: 0.048); how to use medical journals (41% females vs 22%, *p* < 0.001); how to develop culturally appropriate teaching (60 vs 48%, *p*: 0.036); how to work in a community (47 vs 41%, *p*: 0.026); and how to think in new ways about health (45 vs 25%, *p* < 0.001). Where how to understand medical research is concerned there was a difference in level of disagreement (10% females disagree vs 16% males disagree or strongly disagree, *p*: 0.030).

Objective measures of students’ knowledge include student responses to MCQs related to HP topics during the course examinations. As the project has been part of the PP&C3 course since its inception, we do not have data to compare student performance on examinations using a project-based learning approach with a more traditional, lecture-based approach. Examination score averages and course mark averages are comparable with those on other courses in the second year of our program.

Faculty members’ opinions about the project were not taken at this stage. Subjective evaluation by faculty suggests there has been improvement over time in students’ critical thinking and appraisal skills, and in the quality of HP materials. This was apparent from the quality of the projects presented by the students and the topics chosen, as well as by improved oral and written presentation skills. The topics have evolved over the years to cover the country’s key public health priorities. Students’ project materials have shown greater variety, especially where use of social media content to increase the effectiveness of HP messages in reaching the wider community is concerned.

## Discussion

The HP project aimed to improve students’ knowledge and understanding of HP as well as to develop their communication, research, and planning skills. The results of this study showed that most students agreed that the project was helpful to improve their critical thinking, communication skills, research, presentation, and planning skills.

Our use of project-based learning is innovative in this field; as far as we are aware, UAEU is the first university in the UAE or broader region to introduce such a project in the teaching curriculum of a medical program and hence to evaluate its effectiveness and its influence on students. In medical education, there has long been a focus on problem-based learning (30). Team-based learning is also becoming increasingly common (31). Project-based learning is well known in fields such as engineering and education (30, 32), but in medical education it is relatively unknown.

The project is an important introduction to public health early in the medical curriculum. Students receive some basic education and information about public health topics in the first year, and subsequently public health topics are explored in the third and fourth years (Pre-Clinical program) and finally in the fifth year during their public health clerkship rotation (see Supplementary Material). The importance of such integration of HP and prevention education across the medical curriculum and in different preclinical and clinical courses has been emphasized by others (5, 7, 13, 15, 16, 33–37). It is vital to teach physicians different strategies and models of HP to improve their communication with patients and community (9, 38).

Our students have also indicated improvement in planning and research skills. A key element of the HP project is allowing students sufficient time to make mistakes while selecting a topic. Some teams select an appropriate topic on the first day, but others learn much from the requirement to quickly find reliable sources to back up claims that a rather esoteric topic needs HP activity.

Differences between the assessment of the effectiveness of the project between male and female students should be investigated further to establish the reasons. Not only are male and female students taught separately but male cohorts are also smaller. In the three cohorts 2013–2015, male class size was 33, 23, and 22 students, whereas female classes were 99, 114, and 78 students respectively. Average size of male project teams was also smaller. In 2014, when the difference was greatest, there were 5 male teams of 4–5 students, and 16 female teams of 7–8 students. Team size would influence the amount of work individuals must do. The larger number of female teams meant that female students were exposed to a greater variety of HP topics and approaches during the team project presentations. Furthermore, three Medical Education faculty (one for the males, and two splitting the female class) shared the teaching of communication skills, research skills, and project team support, hence the learning experience of males and females was not identical. Finally, sociocultural aspects could play a role. Differences between male and female students’ perceptions of the effectiveness of the project could therefore be related to any of the abovementioned factors.

Improving students’ knowledge about HP will be helpful in disease prevention and in chronic disease management, both of which depend on facilitating change in people’s behavior and lifestyle. Gregg and O’Hara (39) argue that a new system of values and principles is needed to enable practitioners to perform the tasks in contemporary HP. This supports Baum’s definition of the new public health concept (40), which emphasizes the need for more integration of the values and principles of HP in medical curricula to prepare practitioners for the challenges in this field. The approach to HP needs to be holistic and follow a salutogenic approach integrated in all levels of care (41, 42). The focus and design of this project and the students’ positive experience of it indicate the importance and the value of such a modality for improving HP practice in the UAE.

## Acknowledgment of any Conceptual, Methodological, Environmental, or Material Constraints

Although we evaluated the students’ perception of the project, it would be helpful to gather formal feedback from teaching faculty to improve the teaching/learning process, assessment design, and evaluation criteria. We have nevertheless already identified a number of areas where the design or implementation of the project could be improved.

Students learn about HP through their own research for the project, but key topics in HP are introduced in standard lecture format. If faculty are in agreement a flipped classroom approach could be adopted for these sessions to promote more discussion of issues. Team-based learning for this content, while desirable, is precluded by timetabling constraints as effective team-based learning sessions would need longer class sessions than the 45 min available.

The current arrangement of simulated focus group practice exposes students to the general principles of focus groups, but project teams do not get to test their own materials, instead relying on unsystematic feedback from colleagues or faculty. Until 2011, when the HP project was included in the first year of our program, students met with authentic focus groups to evaluate their draft HP materials. When the HP project was moved to year 2, this was discontinued because of logistical and time constraints. Discussion of draft HP materials with appropriate members of the public would improve authenticity of the project task, one of the key criteria for projects in higher education. But as PP&C3 is one of seven courses students are taking, introducing a requirement to arrange a focus group would put undesirable pressure on students. It would be particularly tricky for female students.

Each team of students can freely choose the HP topic to present in their project provided that they find sufficient relevant background information to support their theme. Although prevention remains the focus, students sometimes chose topics based on personal interest, and national public health priorities can be missed. However, if students are unable to show appropriate sources to support their choice of topic, an unusual topic is rejected. This puts such groups under pressure to identify a new topic and complete the project to deadline, but it provides a valuable lesson in the need for reliable evidence in medicine and health care. For this reason, we are reluctant to assign lists of topics for students to select from.

Occasionally students report problems with team dynamics, especially members not participating fully. In such cases, they are advised to try and tackle the problem themselves. In the last 4 years, there was only one team that needed calling in as a group for faculty intervention to help solve problems related to group dynamics and commitment to the task.

Initially there was no proper mechanism to identify whether students had used published sources inappropriately in their HP project materials. Currently SafeAssign (a plagiarism detection tool that is part of the Blackboard virtual learning environment) is used to check text of student produced materials, but more needs to be done to ensure that health education materials developed by students follow copyright ethics, especially where the use of images is concerned. Use of appropriate sources for background material is checked during class project sessions or when groups meet with faculty outside class times. Sources are also cited in the mid-project review and in presentations, but it is possible that poor academic practice is not always identified when assessing project oral presentations in real time.

## Recommendations/Future Directions

Students are producing high quality projects and materials that could well be used by UAE health authorities in their HP campaigns. Those colleagues from outside CMHS who participate in teaching are invited to attend the presentations, but work commitments can make this problematic, therefore in future we should systematically share materials produced. Furthermore, such a project-based learning approach could also be used within other courses: for example, design of clear patient information and education for pharmacology, or production of review materials for other students. This course also potentially offers a platform for collaboration and integration with academic departments in other colleges within the university, for example, mass communication or education.

## Conclusion

Integrating public health and HP practice in the medical curriculum by means of a project-based learning component is an innovative way of encouraging active learning. We offered interactive learning opportunities through student-directed team projects, and this increased student engagement with the area; gave students a solid background in prevention; and led to improved understanding of HP practice, as evidenced by student perceptions of the project as well as evaluation of HP materials produced and examination results. The project also required students to develop and apply research, writing, and presentation skills to an authentic task. The experience of productive team work is also invaluable both for the remaining years in medical school and for their future careers. Such projects could be included in more medical curricula and be tailored to meet the public health needs of the local community, the medical profession, and patients.

## Ethics Statement

This study was carried out in accordance with the recommendations of Ethics guidelines of the CMHS UAE University Ethics Committee. All subjects gave written informed consent in accordance with the Declaration of Helsinki. The protocol was approved by the CMHS UAE University Ethics Committee.

## Author Contributions

All the authors assisted in the interpretation of data and manuscript drafting; reviewed final version.

## Conflict of Interest Statement

The authors declare that the research was conducted in the absence of any commercial or financial relationships that could be construed as a potential conflict of interest.
