# Supplementary figures and images for: Integrating Public Health and Health Promotion Practice in the Medical Curriculum: A Self-Directed Team-Based Project Approach
Source: Front Public Health. 2017 Aug 21;5:193. doi: 10.3389/fpubh.2017.00193 (PMC5573437; doi:10.3389/fpubh.2017.00193)

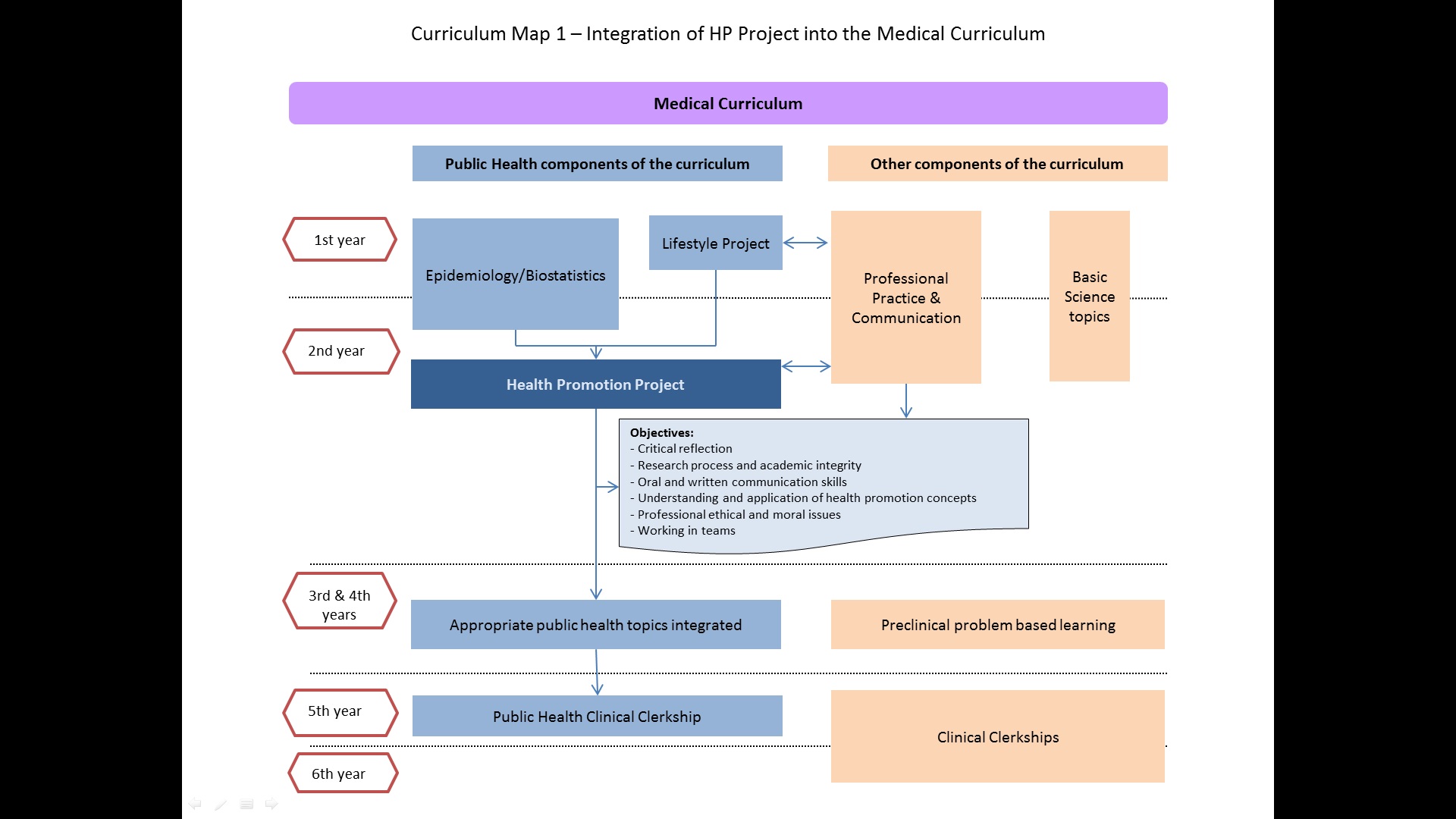

Supplement: Supplementary file 1 [file Image_1.JPEG]
